# Climate Change Impacts on Sunflower (*Helianthus annus* L.) Plants

**DOI:** 10.3390/plants10122646

**Published:** 2021-12-01

**Authors:** Eloísa Agüera, Purificación de la Haba

**Affiliations:** Department of Botany, Ecology and Plant Physiology, Faculty of Science, University of Córdoba, 14071 Córdoba, Spain; bv1hahep@uco.es

**Keywords:** carbon metabolism, growth, nitrogen metabolism, oxidative state, photosynthesis

## Abstract

The biochemical, biological, and morphogenetic processes of plants are affected by ongoing climate change, causing alterations in crop development, growth, and productivity. Climate change is currently producing ecosystem modifications, making it essential to study plants with an improved adaptive capacity in the face of environmental modifications. This work examines the physiological and metabolic changes taking place during the development of sunflower plants due to environmental modifications resulting from climate change: elevated concentrations of atmospheric carbon dioxide (CO_2_) and increased temperatures. Variations in growth, and carbon and nitrogen metabolism, as well as their effect on the plant’s oxidative state in sunflower (*Helianthus annus* L.) plants, are studied. An understanding of the effect of these interacting factors (elevated CO_2_ and elevated temperatures) on plant development and stress response is imperative to understand the impact of climate change on plant productivity.

## 1. Introduction

The UN Framework Convention (1992) on Climate Change defines climate change as a type of climate modification that is attributed directly or indirectly to human activity. This modification alters the composition of the global atmosphere and acts in addition to the natural climate variability, which can be observed over comparable time periods.

**Figure 1:**
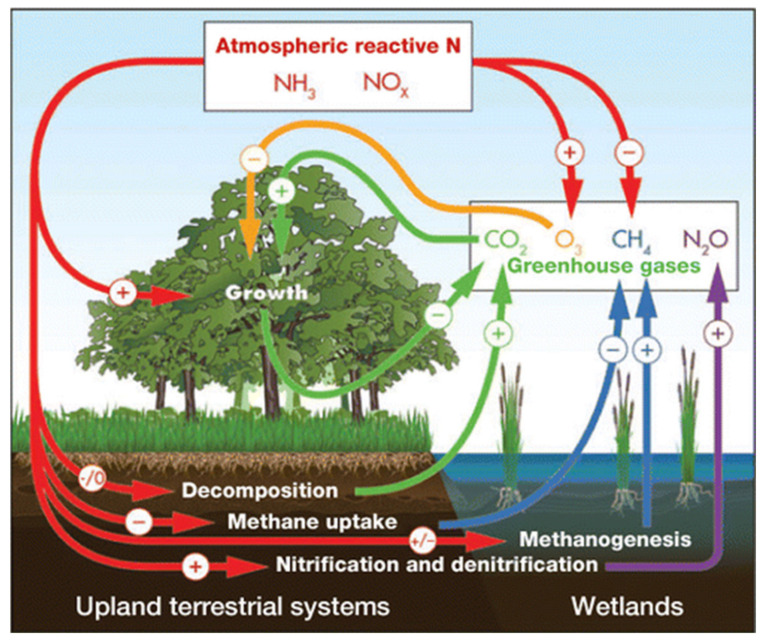
Gases and processes involved in the greenhouse effect. (Sources from Templer et al. 2012).

During the last decades, anthropic emissions of greenhouse gases, such carbon dioxide (CO_2_), nitrous oxide (N_2_O), and methane (CH_4_) have induced alterations in natural climate cycles of the Earth, elevating the mean surface temperature of the planet [1]. The Intergovernmental Panel on Climate Change (IPCC) predicted that between 2060 and 2100, CO_2_ levels will reach concentrations of 660–790 μL L^−1^, while the global surface temperature will be between 2.0 and 3.7 °C above the pre-industrial average temperature [2]. Ongoing gas emission is one of the current causes of climate change, since it leads to increased temperatures due to gas absorbing infrared radiation [3,4]. On the other hand, the intensive use of chemical fertilizers alters the global cycle of nitrogen, increasing the levels of N_2_O and NO, which also promotes global warming [5] (Figure 1).

Climate change causes major alterations in ecosystems, leading to extreme climate-related phenomena, such as droughts, floods, heatwaves, hurricanes, etc. [2]. In general, the biochemical, biological, and morphogenetic processes of plants are affected by climate change, resulting in alterations in their development, growth, and productivity [6]. The decrease in plant performance is mainly caused by biotic and abiotic stress factors. Attaining new stress-resistant crops is a priority for both conventional and modern improvement (biotechnological). Gruissem et al. [7] suggested the importance of studying plants that are more flexible and have a greater adaptive capacity with respect to the modifications produced by climate change.

Sunflower, the fourth most important oil crop worldwide, is normally susceptible to low temperatures and salinity [8,9,10]. The sunflower crop is a rainfed crop, showing tolerance to water stress conditions by presenting a highly explorative root system [11,12,13]. Although the mechanisms involved in this tolerance remain unclear at the molecular level, an increase in the expression level of photosynthesis related genes as well as higher levels of sugars, osmoprotectant amino acids, and ionic nutrients under water stress conditions have recently been observed in sunflower plants. In addition, transcription factors have been identified that were upregulated during water stress conditions and that may act as hubs in the transcriptional network. Many of these transcription factors belong to families implicated in the water stress response in model species [14]. These findings will provide useful biotechnological tools to improve stress tolerance while maintaining crop yield under restricted water availability.

Therefore, this review focuses on the physiological and metabolic changes taking place during the development of sunflower plants due to environmental modifications resulting from climate change, especially elevated concentrations of atmospheric carbon dioxide (CO_2_) and increased temperatures.

## 2. Effects of Elevated CO_2_ and Elevated Temperatures on Sunflower Plants Growth

In general, elevated CO_2_ levels, directly and indirectly affect plant growth and development, modifying numerous physiological processes. Elevated concentrations of CO_2_ tend to increase plant growth and produce large quantities of biomass, especially C3 plants, since they provide additional C (fertilization effect) [15]. Plant growth is determined by cell division and expansion. These processes are coordinated and controlled during organogenesis though a series of factors, including vegetable hormones, and they respond to environmental signals [16,17,18]. An elevated atmospheric CO_2_ concentration level may positively influence cell division and expansion [19,20]. Increased cell expansion is associated with greater extensibility of the cell wall and increased activity of the enzymes that fluidify the wall, such as xyloglucan endotransglucosylase (XET) [21]. It has been found that in soy leaves and *Betula papyrifera,* which are grown in a CO_2-_enriched environment, certain genes participating in the cell cycle (coding histones) or fluidifying the cell wall (coding expansins and XET) increase their expression [22,23]. It has been verified that a major supplement of carbon at elevated CO_2_ concentrations may contribute to accelerating cell division and expansion in meristematic tissues and improves early plant growth and development [24]. Sunflowers grown at elevated CO_2_ concentrations were shown to reveal improved growth, reflected in an increased specific leaf mass (SLM), which refers to the dry weight of young leaves (16 days) [25]. It is unclear whether or not this increased cell cycle activity resulting from the increased CO_2_ is due to the fact that the plant has more photoassimilates for growth or whether it is because of the divergence produced in gene expression in response to the increased sugar levels [26]. However, in sunflower plants grown at elevated temperatures, a reduced growth has been observed, as reflected when determining the SLM and area of the leaf as well as the soluble protein content [27]. Elevated temperatures negatively affect cell division as well as cell expansion since temperature is one of the main stresses stimulating protein degradation and causing tissue senescence or death [28,29]. Elevated CO_2_ stimulates the root and shoot growth of wheat, but this stimulation was found to reduce when plants were grown in combined elevated temperature and elevated CO_2_ [30]. Lee et al. [31] showed that while elevated temperatures may negatively influence the growth and yield of potato crops, concurrent and appropriate elevation of CO_2_ and temperature can promote balanced development of source and sink organs and positively affect potato productivity. Field experiments on sunflower production using an OilCROP-SUN model predicted that the increase in temperature negatively affects sunflower productivity in Pakistan. Although increased CO_2_ concentration showed a positive effect on sunflower, it does not fully compensate for the negative effect of increased temperature [32]. In irrigated crops, adaptation to climate change depends on the availability of water, thus the combined effects of high atmospheric CO_2_ and climate change decrease crop yields if agricultural management practices are not modified [33].

## 3. Elevated CO_2_ Levels and Elevated Temperatures on Carbon Metabolism in Sunflower Plants

Elevated levels of CO_2_ increase the photosynthetic rate; therefore, crop growth and productivity are increased [34]. It has been observed that an elevated concentration of CO_2_ stimulates the photosynthetic fixation of CO_2_, as well as stoma transpiration and conductance in young sunflower plant leaves [25]. Elevated levels of CO_2_ concentration increase the photosynthesis rate in C3 plants, since the Ribulose-1,5-bisphosphate carboxylase/oxygenase (Rubisco) enzyme involved in the fixation process of CO_2_ and photorespiration, is not saturated in the environmental CO_2_ concentration [35]. Therefore, an increase in atmospheric CO_2_ would increase the leaf’s level of internal CO_2_, as well as the CO_2_/O_2_ ratio, affecting the Rubisco and thereby favoring the carboxylation reaction as compared with the oxygenation process. Elevated CO_2_ concentrations may reduce the photorespiration process in C3 plants and, therefore, the production of cellular hydrogen peroxide (H_2_O_2_) derived from the metabolism of glycolate [36,37]. On the other hand, it has been shown that the efficiency of photosystem I and II (PSI and PSII) increases at elevated levels of CO_2_, producing more adenosine triphosphate (ATP) and reduced nicotinamide adenine dinucleotide phosphate (NADPH) [38,39]. In addition, increased efficiency in the use of light is observed as a result of the increased flow of electrons between the PSII and PSI under circumstances of high CO_2_ [40]. Vicente et al. [41] revealed an increased gene and protein expression related to light reactions of photosynthesis.

This stimulating effect of photosynthesis caused by elevated levels of CO_2_ may be temporary, given the acclimation of photosynthesis to elevated concentrations of CO_2_, which initially stimulates the fixation of C but is followed by a slow decrease in the C fixation process [42]. Various studies have indicated that the acclimation of photosynthesis is due to factors such as reduced content of Rubisco [43], the inhibition of the assimilation of C due to the accumulation of non-structural carbohydrates that suppress the expression of genes related to photosynthesis [43,44], and a reduction in the concentration of nutrients, especially N in plant tissues, due to the inhibition of photoassimilates of NO_3_^−^ [45,46,47]. In *Populus tremuloides* and *B. papyrifera* in the presence of elevated CO_2_, net photosynthesis increased by 43–73% and the hexose ratio increased when compared with that of sucrose [48]. This was also observed in sunflower leaves [25]. When cucumber plants were grown at high concentrations of CO_2,_ an increase in the content of starch and soluble sugars was also observed in the leaf, as well as a decrease in the content of nitrogen [49,50]. However, the effect of the elevated CO_2_ on the accumulation of hexose varied between species [51,52], as did the sensitivity of the distinct plant tissues [53].

The plant growth and yield depend upon the species specific temperature optimum [54]. An elevated temperature conditions the rate of enzymatic reactions and modifies the structure and activity of macromolecules [55]. In addition, it is known that elevated temperatures modify the composition and structure of cell membranes, increasing the fluidity of membrane lipids and decreasing electrostatic interactions between polar groups of the proteins within the aqueous phase of the membrane and producing a loss of ions [56]. Therefore, photosynthesis at elevated temperatures is modified, since the thylakoid membrane is altered along with the thylakoid shape and arrangement [57]. On the other hand, high temperatures also cause photoinhibition of the PSII through the effect on the oxygen emitter complex, which is destroyed by heat [58,59,60]. The decreased photosynthetic rate may also be due to the fact that elevated temperatures cause stomatal closure to prevent water loss, resulting in a decreased exchange of gases between the leaf and the atmosphere [61]. De la Mata et al. [27], attributed the lower net photosynthesis to elevated temperatures in primary sunflower leaves, compared with a control group, causing a reduction in photosynthetic pigments and partial stomatal closure. Greer and Weedon [62] observed that the average rates of photosynthesis of *Vitis vinifera* leaves decreased by 60% when temperatures increased from 25 to 45 ºC. This reduction in photosynthesis was attributed to 15–30% stomatal closure. The photosynthetic rate is also determined by the capacity of carboxylation of Rubisco, which is highly dependent on temperature. Elevated temperatures decrease the state of activation of Rubisco due to the inactivation of the Rubisco activase enzyme, thereby affecting the carbamylation process of the Rubisco [63,64,65,66]. When Rubisco acts as carboxylase, products are frequently formed that prevent its activation, and these should be eliminated from the active site by the Rubisco activase [67,68]. Rubisco activase is relatively labile to heat [65,69]; therefore, its capacity to maintain the Rubisco’s state of activation is expected to decrease with elevated temperatures. Plants expressing a more thermotolerant Rubisco activase have higher net photosynthesis at elevated temperatures [70,71]. On the other hand, as the temperature increases, the rate of photosynthesis decreases, with the rate of photorespiration increasing more rapidly [72]. There are two reasons for this. First, as temperatures increase, Rubisco’s affinity for CO_2_ decreases compared with that of the O_2_. Thus, the oxygenation reaction of the Rubisco is more frequent [73,74]. Second, as the temperature increases, the O_2_ solubility decreases more slowly than the CO_2_ solubility [75]. Therefore, in warm environments, there is relatively more O_2_ available to react with the Rubisco.

The clearest evidence that elevated CO_2_ and elevated temperatures will alter plant carbon fluxes comes from studies that manipulate both factors [46,76]. These data imply that the plant carbon flux response to temperature varies across species. Lee et al. [31] observed that the concurrent elevation of temperature and CO_2_ enhanced plant thermostability and reduced the damaging effect of elevated temperatures in potato plants.

## 4. Elevated CO_2_ Levels and Elevated Temperatures on Nitrogen Metabolism in Sunflower Plants

Nitrogen is the mineral with the greatest impact in terms of limiting the primary growth and productivity of plants in natural systems and in agriculture. In most soils, nitrogen tends to appear in the form of nitrate (NO_3_^−^), since ammonium (NH_4_^+^), including that which is added to the soil as fertilizer, is rapidly oxidized to NO_3_^−^ by nitrifying bacteria. In plants, nitric nitrogen converts into ammonium nitrogen, a process known as assimilatory reduction in NO_3_. The assimilation of NO_3_^−^ is regulated by endogenous and/or exogenous factors, such as NO_3_^−^, carbon compounds, and light. NH_4_^+^ produced from the assimilatory reduction in NO_3_^−^, combined with that resulting from other metabolic reactions, is added to the carbon compounds to synthesize nitrogenated compounds that the plant uses for its growth [77].

Stitt and Krapp [78] initially assumed that some plant species required a higher rate of NO_3_^−^ assimilation to permit increased plant growth under conditions of elevated CO_2_ concentrations. However, it was found that CO_2_ enrichment inhibits the assimilation of NO_3_^−^ in sunflowers [79] as well as in wheat plants, *Arabidopsis* [80], and field-grown wheat [45]. The assimilation of NO_3_^−^ requires the reduced form of nicotinamide adenine dinucleotide (NADH) in order for the nitrate reductase (NR) to catalyze the formation of NO_2_^−^ based on NO_3_^−^_._ Photorespiration stimulates the release of malate from the chloroplasts and increases the availability of NADH in the cytosol, thereby increasing the NR activity [81], which permits the first step in NO_3_^−^ assimilation [82]. Elevated CO_2_ concentrations reduce photorespiration and thus, decrease the quantity of NADH available for the reduction in NO_3_^−^_._ This may explain the decreased levels of NR activity observed in sunflower plants under conditions of elevated CO_2_ [79]. However, six transporters from the Nar1 family are involved in the translocation of NO_2_^–^ from the cytosol to the chloroplast in *Chlamydomonas* some of these transport both NO_2_^–^ as well as HCO_3_^–^ [83]. Bloom et al. [84] revealed that HCO_3_^−^ inhibits the entry of NO_2_^–^ in isolated chloroplasts of wheat and peas, indicating that an analogous system is operating in higher plants. Therefore, a decrease in the affluence of NO_2_^−^ to the chloroplast may result from higher CO_2_ levels, which may also explain the reduced glutamine synthetase (GS) activity observed in sunflower plants grown under enriched CO_2_ conditions [79]. Studies have shown that both chloroplast isoforms and GS cytosols are affected by abiotic stress [85]. De la Mata et al. [79] observed that an elevated CO_2_ level significantly increased the relative expression of the GS1 isoform (cytosol), but decreased the GS2 transcription levels (chloroplast) in sunflower leaves. Recently, a high correlation was reported between increases in carbohydrate content and the downregulation of genes involved in photosynthesis and N metabolism [86].

In sunflower plants grown at elevated CO_2_ levels (800 μL L) and elevated nitrate availability (25 mM), the primary leaves reveal an increased growth, photosynthetic capacity, assimilation of nitrogen, and antioxidant defenses compared with plants grown at elevated CO_2_ levels and limited nitrogen. This results in a delay in the leaf’s senescence process, demonstrating that the induction of the senescence process is directly related to the C/N ratio of the leaf [87]. This C/N ratio should be balanced in order for the plant to be more productive. An elevated CO_2_ increases this ratio in plants due to the decrease in nitrogen content in the leaf [79]. Sunflower plants that are biofertilized via inoculation with mycorrhizal fungi (*Rhizophagus irregularis*) and are grown in environments of elevated CO_2_, and reveal a decrease in the C/N ratio compared with plants grown at elevated CO_2_ levels and without biofertilizers. These results suggest that sunflower symbiosis with *R. irregularis* improves the absorption of nitrogen favoring the stability of the C/N ratio in the plant, despite the elevated atmospheric CO_2_ levels [88]. De la Haba et al. [27] observed in primary leaves of sunflower plants that the NR and GS activity decreased while decreased activity in glutamate dehydrogenase increased in leaves exposed to elevated temperature. A superior decrease in the soluble protein content during leaf life span in plants grown at elevated temperatures suggests that elevated temperatures promote soluble protein degradation in sunflower leaf.

Although elevated CO_2_ concentrations and temperatures have been treated separately, in terms of the reduced availability of nitrogen in plants, especially sunflowers [16,18], there are little data on the combined effect of these factors. More research is necessary before any broad-scale conclusions can be made with regard to the interaction between elevated CO_2_ and elevated temperature.

## 5. Oxidative Stress in Sunflower Plants to Elevated CO_2_ and Temperature

Elevated levels of CO_2_ decreased the content of photosynthetic pigments (chlorophyll a and b and carotenoids) and increased the oxidative stress on the sunflower plants, by increasing H_2_O_2_ levels and decreasing the activity of antioxidant enzymes, such as catalase and ascorbate peroxidase [25]. The loss of antioxidant defenses in the plant probably increases the concentration of the reactive species of oxygen in the chloroplast, thereby decreasing the content of photosynthetic pigments [25]. Biofertilization through fungi (*Rhizophagus irregularis*) in sunflower plants grown in environments of high CO_2_ reveals a decreased hydrogen peroxide content and increased antioxidant enzyme activity (catalase and APX). These results suggest that sunflower symbiosis with *R. irregularis* decreases the plant’s oxidative stress [88]. Seemingly, an increase in antioxidant defenses is a mechanism that can be used to mitigate the effect of CO_2_ on plants [89]. A better understanding of these processes during leaf development is essential to improving crop productivity in a CO_2_-rich atmosphere. It has also been observed that elevated temperatures decrease activity levels of antioxidant enzymes [90] and induce oxidative stress in plants since reactive oxygen species (ROSs) are produced, for example, superoxide radicals (O_2_−), H_2_O_2,_ and hydroxyl radicals (HO˙) [91]. The accumulation of ROS not only has negative consequences on cells but also acts on the stress signaling pathways, activating the synthesis of thermal shock protein transcription factors [92]. It was suggested that, similar to other types of abiotic stress, stress caused by heat may decouple enzymes and metabolic channels that cause an accumulation of ROSs, which are responsible for oxidative stress [93]. In sunflower plants grown at elevated temperatures, considerable oxidative stress was found during leaf development, as revealed by the significant increase in H_2_O_2_ and the clear decrease in antioxidant enzyme activity (CAT and APX) compared with plants grown at control temperatures [27]. An increased expression of CAT and APX at elevated temperatures in heat-tolerant sugar cane leaf genotypes may protect from ROSs and H_2_O_2_, superoxide and hydroxyl radicals caused by plants exposed to high temperatures [94]. Elevated temperatures decrease the growth of 42-day-old sunflower primary leaves, negatively affecting markers that are commonly used to monitor leaf development and increasing oxidative state 42-day-old sunflower primary leaves [95].

It has recently been observed that the protein profiles examined in sunflower leaves revealed marked differences in protein expression between plants grown under the two temperature conditions (low and elevated temperatures). Interestingly, 26.4% of the identified proteins, mainly categorized in four functional groups (1-antioxidant, 2-stress and defense, 3-energy and metabolism-related, and 4-hormonal regulation proteins), exhibited increased expression in response to higher growth temperatures. These molecular differences detected in primary leaves at elevated temperatures can indicate a greater tolerance of sunflower plants to these stress conditions [95].

## 6. Conclusions

Within the context of current environmental conditions and those projected for the coming decades, an urgent need exists to increase crop performance by developing crops that are resistant to environmental changes. We believe that deepening our understanding of the combined effects of increased temperatures and CO_2_ concentrations on the development of sunflower plants is essential to predict the impact of climate change because the sunflower is an important oil crop worldwide. Therefore, we advocate the expansion of studies in sunflower plants, combining elevated CO_2_ and elevated temperature to provide the information required to guide strategies that provide plant improvement in a future climate. Emphasizing the need to address the responses of growth, carbon and nitrogen metabolism, as well as the oxidative state of the plant to climate change, will provide comprehensive information and open new pathways to mitigate and adapt to the impacts of increased CO_2_ and temperatures in vegetation.

**Figure 2:**
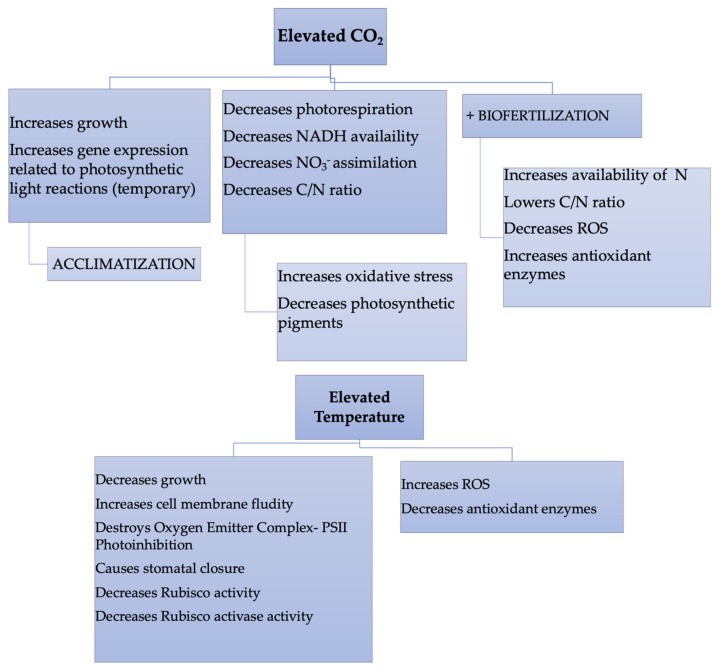
Modifications in sunflower plants due to the increase in CO_2_ and environmental temperatures.

Figure 2 shows a summary of the modifications taking place in sunflower plants when grown to elevated CO_2_ levels and temperatures independently, a result of the different investigations carried out by our group on sunflowers.

## Data Availability

Not applicable.
